# PHYSICAL FUNCTIONING 12 YEARS BEFORE AND AFTER KNEE OR HIP SURGERY IN OLDER WOMEN

**DOI:** 10.1093/geroni/igad104.0374

**Published:** 2023-12-21

**Authors:** Geeske Peeters, Isabel de Munck, Leigh Tooth, Rene Melis

**Affiliations:** Radboud University Medical Center, Nijmegen, Gelderland, Netherlands; Radboud university medical centre, Nijmegen, The Netherlands, Nijmegen, Gelderland, Netherlands; The University of Queensland, Brisbane, Queensland, Australia; Radboud University Medical Center, Nijmegen, Gelderland, Netherlands

## Abstract

Understanding long-term recovery patterns in the context of ageing-related decline may improve knee or hip surgery outcomes. We examined the impact of hip and knee surgery on long-term trajectories of physical functioning (PF) in older women, and profiled women with higher and lower resilience to surgery. Resilience, defined as bouncing back (PF) after a stressor (hip or knee surgery), was quantified using the expected recovery differential method. Data were from 10,434 women (73-79 yrs) in the Australian Longitudinal Study on Women’s Health who completed surveys 2-6 (1999-2011). Adjusted linear mixed models were run to examine the surgery-by-time (-12 to +12 yrs) interaction in association with PF (SF-36 subscale). The difference between observed and expected PF were calculated, with positive/negative values reflecting higher/lower resilience, respectively. PF trajectories differed between women with (n=982) and without (n=8117) hip surgery (p<0.001). Among hip surgery patients, the decline was more rapid pre-surgery than post-surgery (b=-0.7, p<0.001). Women with knee surgery (n=1144) had lower PF than those without surgery (n=7971), but with a slower rate of decline (p=0.01). Among knee surgery patients, the rate of decline was similar pre- and post-surgery (b=-0.3, p=0.25). Both in hip and knee patients, women with higher resilience had fewer comorbidities and symptoms, and were more often physically active and independent in daily activities than those with lower resilience (p<0.05). In conclusion, women with and without hip or knee surgery have different trajectories of PF. Women who function better than expected after surgery, tend to have fewer health problems.

